# New Insights into Functional Roles of the Polypyrimidine Tract-Binding Protein

**DOI:** 10.3390/ijms141122906

**Published:** 2013-11-20

**Authors:** Maria Grazia Romanelli, Erica Diani, Patricia Marie-Jeanne Lievens

**Affiliations:** Department of Life and Reproduction Sciences, Section of Biology and Genetics, University of Verona, Strada Le Grazie 8, 37134 Verona, Italy; E-Mails: erica.diani@univr.it (E.D.); patricia.lievens@univr.it (P.M.-J.L.)

**Keywords:** PTB, raver1, alternative splicing, IRES, RRM, RNA binding proteins, ribonucleoproteins, hnRNP, mRNA stability, cell differentiation

## Abstract

Polypyrimidine Tract Binding Protein (PTB) is an intensely studied RNA binding protein involved in several post-transcriptional regulatory events of gene expression. Initially described as a pre-mRNA splicing regulator, PTB is now widely accepted as a multifunctional protein shuttling between nucleus and cytoplasm. Accordingly, PTB can interact with selected RNA targets, structural elements and proteins. There is increasing evidence that PTB and its paralog PTBP2 play a major role as repressors of alternatively spliced exons, whose transcription is tissue-regulated. In addition to alternative splicing, PTB is involved in almost all steps of mRNA metabolism, including polyadenylation, mRNA stability and initiation of protein translation. Furthermore, it is well established that PTB recruitment in internal ribosome entry site (IRES) activates the translation of picornaviral and cellular proteins. Detailed studies of the structural properties of PTB have contributed to our understanding of the mechanism of RNA binding by RNA Recognition Motif (RRM) domains. In the present review, we will describe the structural properties of PTB, its paralogs and co-factors, the role in post-transcriptional regulation and actions in cell differentiation and pathogenesis. Defining the multifunctional roles of PTB will contribute to the understanding of key regulatory events in gene expression.

## 1. Introduction

The control of gene expression is a fundamental process required by all living cells. In eukaryotic cells, multiple and coordinated processes contribute to the fine-tuning of the transcription and translation of selected genes in the nucleus and the cytoplasm. The initial steps take place in the nucleus where promoter recognition, mediated by transcription factors, requires chromatin and nucleosome modifications driven by epigenetic events, and allows the regulation of the RNA polymerase transcriptional activity. When pre-mRNAs emerge from the transcription sites, they are associated with *trans*-acting proteins and RNAs, to form the RNA-protein complexes also referred as ribonucleoprotein (RNP) complexes.

**Figure 1:**
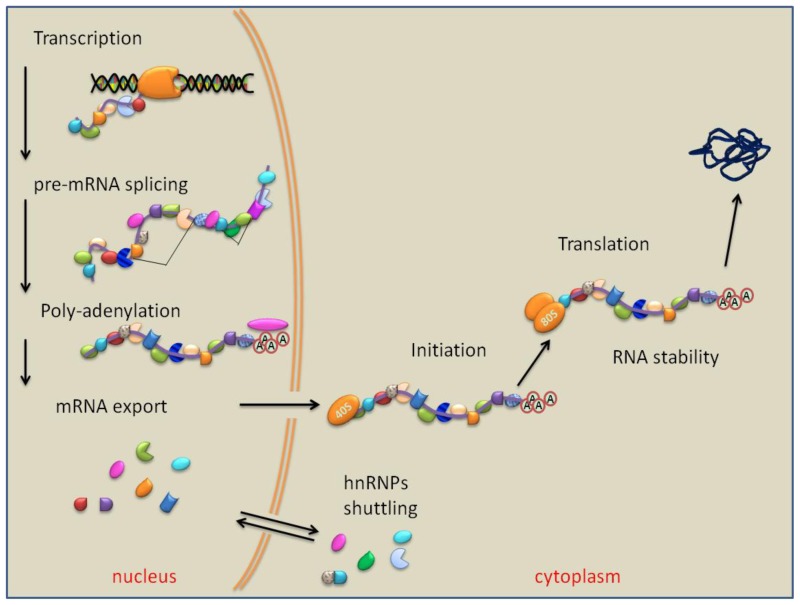
RNA binding proteins are involved in all steps of RNA biogenesis. Starting from pre-mRNA transcript synthesis, a variety of different RNA binding proteins are associated within the heterogeneous ribonucleoprotein complexes, participating in the processes which result in the formation of mature mRNA, including pre-mRNA splicing and addition of 3′ poly(A) tails in the nucleus and mRNA export through the nuclear pores. In the cytoplasm, they participate in localization, initiation of translation, and mRNA stability.

The RNPs assembled on each mRNA diversify their composition by recruiting and reassorting selected components. During mRNA maturation, the RNPs are further recombined and modelled, regulating the subsequent stages of RNA biogenesis, which include the 5′-end capping, splice site recognition, RNA editing, 3′-end cleavage, polyadenylation, nuclear pores export, sub-cellular localization, ending to protein translation and RNA degradation [1–3] (Figure 1). Alternative splicing (AS) of the precursor mRNA (pre-mRNA) occurs in >90% of human genes and represents the principal mechanism responsible for the complexity of genome expression and protein diversity [4–6].

Two major RNA binding protein families, the heterogenous nuclear ribonucleoproteins (hnRNPs) and the serine/arginine (SR)-rich proteins (SR proteins) contribute to AS events within RNP complexes [7–10]. Both families of RNA binding proteins are associated with nascent transcripts and influence most steps of mRNA biogenesis by synergic or antagonist effects on similar processes [11]. Typical SR proteins bind to enhancers of exon splicing, activating constitutive and alternative splice sites by recruiting spliceosomal components to the pre-mRNA [12]. Most of these splicing factors are ubiquitously expressed, but their relative abundance or activity may vary in the tissues and can be modulated by the induction of specific cellular signaling pathways [13–15]. HnRNPs, instead, influence constitutive and alternative splicing events by preferentially binding to splicing silencers [12]. The hnRNPs form ribonucleoprotein complexes that are distinct from small nuclear ribonucleoprotein particles (snRNPs) of the spliceosomal complexes. The combination of different types of hnRNPs in the ribonucleoprotein complexes bound to the pre-mRNA may, in turn, antagonize or favor splice site selection. Their action supports the structural modeling of pre-mRNA, bringing together regions that are distant hundreds of nucleotides, and assists in the splice site recognition [16].

One of the most investigated exon recognition repressors, belonging to the hnRNPs family, is the polypyrimidine tract-binding protein (PTB) that binds to intron pyrimidine-rich elements and mediates tissue-specific regulation of exon splicing [17,18].

PTB is recruited not only in AS, but in additional steps of RNA processes eliciting various biological effects, including transport, stabilization, and mRNA translation. The cell type and the position of the specific RNA binding sites are determinant factors for the variety of PTB effects on RNA destiny [19]. Starting from its first description in 1992 [20,21], an increasing number of studies have been published, describing structural and functional properties of PTB, which thus became one of the most cited proteins in studies focusing on nuclear localization, alternative splicing, and RNA binding proteins.

This review will focus on the established roles of PTB in splicing regulation and protein translation highlighting the most recent contributions to the understanding of its multi-functionality.

## 2. *PTB* Gene Expression

*PTB* (accredited gene name *PTBP1*), also known as *hnRNP I*, is a shuttling protein that moves rapidly between nucleus and cytoplasm, with a predominant nuclear localization [22]. The 57 kDa protein is organized into four RNA recognition motifs (RRM1–4), a bipartite nuclear localization determinant (NLD), which we have demonstrated to be recognized by importin α [23,24], and a nuclear export signal at the *N*-terminus of the protein [25]. The *PTB* gene maps on chromosome 19 at position p13.1, and is organized in 15 exons that are subjected to alternative splicing, producing at least four isoforms [26]. PTB1 is the first described isoform and consists of 521 amino acids containing all four RRMs. The alternatively spliced isoforms, PTB3 and PTB4, contain additional 19 or 26 amino acids between the RRM2 and RRM3 domains derived from exon 9 inclusion, which has two alternative 3′ splice sites. The alternative splicing of exons 2–10 produces the fourth isoform, named PTB2, which is translated in a protein lacking the RRM1 and RRM2. PTB is expressed in all human tissues in different isoforms and levels according to the specific cell type [26]. In spite of their high levels of homology, the different PTB proteins show distinct activities in splicing and in IRES-mediated initiation of translation [19].

## 3. Unique Properties of PTB RNA Recognition Motifs

**Figure 2:**
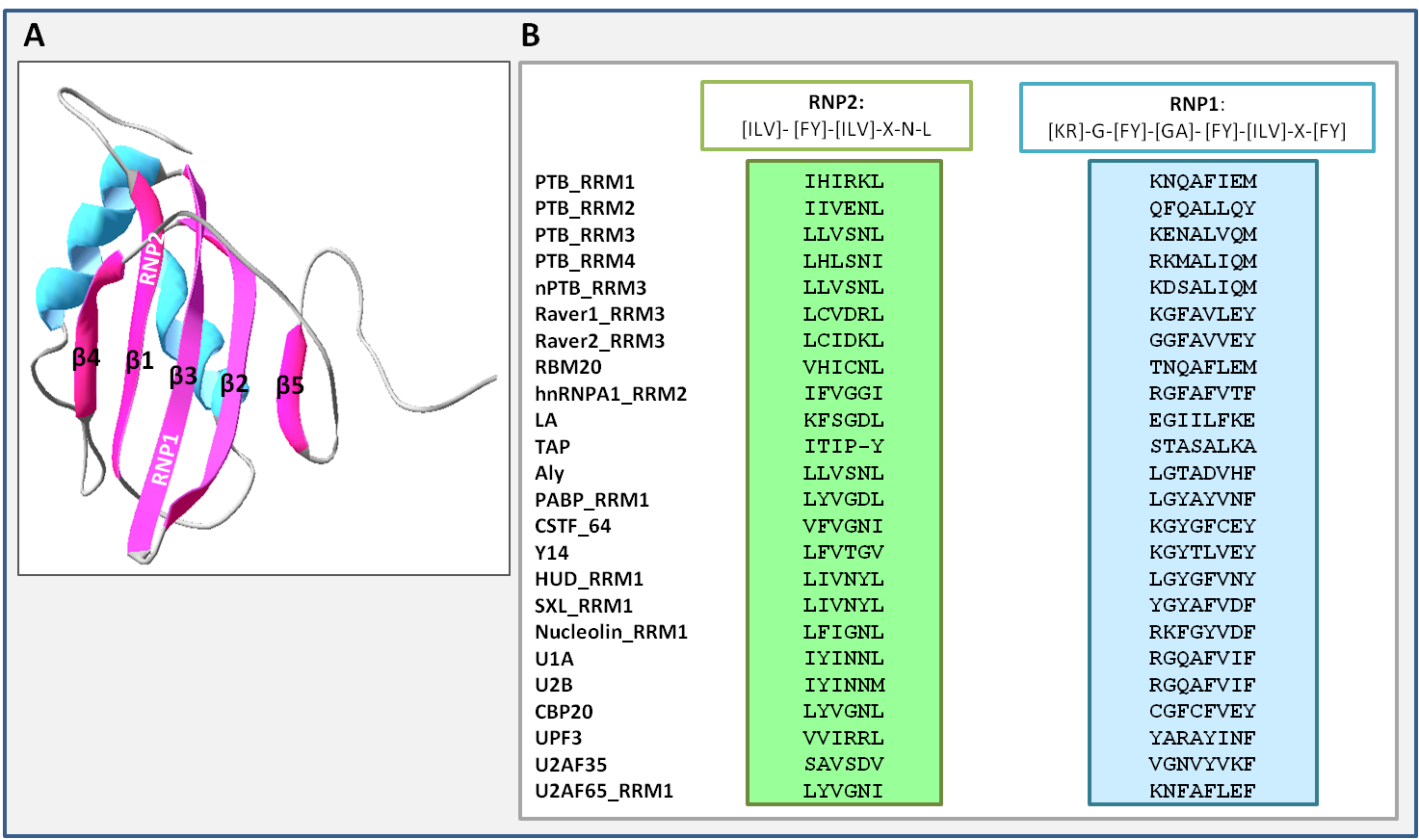
PTB RRM3 structure. (**A**) The PTB RRM3 structure was obtained with Swiss-PdbViewer (http://www.expasy.org/spdbv/); (**B**) Alignments of ribonucleoprotein (RNP) 2 and RNP1 sequences present in the PTB RRM3 with RRM containing proteins. The alignment was generated by ClustalW and manually optimized. PTB, polypryrimidine tract binding protein; nPTB, neuronal PTB; hnRNPA1, heterogeneous nuclear ribonucleoprotein type A1; LA, La protein; TAP, mRNA export factor Tap; PABP, Poly(A)-binding protein; CSTF64, cleavage stimulation factor 64; Y14, Y14 Magoh complex; HUD, Hu protein; SXL, Sex-Lethal; U1A, U2B, U1 snRNP proteins; CBP20, cap-binding protein 20; UPF3, up-frameshift protein 3; U2AF65, U2 snRNP auxiliary factor; RBM20, RNA binding motif 20.

The members of the hnRNP protein family were initially discovered in the late 1980s and were attributed to the same protein family by the presence of the RRM, which is the most conserved domain among the RNA binding domains (RBDs). The RRM is a functional domain broadly distributed in hundreds of proteins encoded by eukaryotic genomes. Compared to other known RBDs, such as the K-homology domain, the RGG (Arg-Gly-Gly) box, the Sm domain, the DEAD/DEAH box, the Pumilio/FBF (PUF domain) and the Piwi/Argonaute/Zwille (PAZ), for review see [27], RRM is structurally quite different. RRMs containing genes represent about 0.5%–1% of the human genes. The RRM is often present in more than one copy and can be associated with additional protein domains, e.g. zinc finger of the CCCH type, the polyadenylate binding protein *C*-terminal domain (PABP), and the WW domain [28]. The structural and biochemical properties of the RRMs have contributed to the interpretation of RNA recognition capability, but also to the identification of protein-protein interactions between splicing factors [29,30]. Canonical RRMs consist of approximately 90 amino acids, containing two conserved core sequences, named RNP2 and RNP1, directly involved in the RNA interaction (Figure 2A) [28]. In a typical RRM, the peptide folds with a β1α1β2β3α2β4 topology leading to a structural model of four β-sheets packed against two α-helices (Figure 2A). Both RRM1 and RRM4 domains of PTB fold in the canonical βαββαβ RRM topology, whereas RRM2 and RRM3 contain an additional fifth β-strand (Figure 2A) that extends the canonical RNA binding surface [31,32] and contributes to sequence specific recognition.

RRM3 can compete with the pre-mRNA splicing factor U2AF65 (U2 auxiliary factor) at polypyrimidine stretches located at the 3′ splice site of target introns [33]. In addition to the presence of a fifth β-strand in RRM2 and RRM3, all four RRMs present in PTB show divergences from the conserved RRM sequences, represented by the absence of the aromatic side chains typically found in RNPs (Figure 2B).

The peculiarity of the structural features of the RRMs allows PTB to be involved in the splice site choice in alternative splicing events, but also to be recruited in several additional events in post-transcriptional regulation, including RNA stability and translation. The consensus sequences for RNA-PTB binding are represented by sequences containing 15–25 pyrimidines, with a preference for pyrimidine tracts containing UCUU, CUCUCU [34–36]. The structures of PTB complexed with RNA have been intensively investigated by NMR spectroscopy [37]. The solved three dimensional solution structures demonstrate that each RRM of PTB may bind one RNA molecule containing the CUCUCU sequence, and all RRMs bind polypyrimidine tracts by the contribution of their β-sheets. While RRMs 1 and 2 remain independent in solution, RRMs 3 and 4 may interact with each other producing a single globular protein moiety. RRM3 and 4 bind to pyrimidine tracts separated by intervening sequences of 15 nucleotides or longer, inducing RNA looping [37–39].

RRM1 and RRM2 are separated by flexible linkers from the rest of the protein, which allow them to adopt different conformations and to favour contact with specifically structured RNA. A novel interaction with U1 snRNA has been recently attributed to RRM1 and RRM2 and this represents the first demonstration of a direct interaction between PTB and the spliceosome [40]. Detailed analyses described by Clerte and Hall [41] and Kafasla *et al.* [42], have highlighted a modular function of PTB-RNA binding in which RRM1 and RRM2 preferentially bind to short tracts of U/C located in loop structured RNA sequences, whereas the interacting RRM3 and RRM4 preferentially bind to longer flexible RNA sequences.

In addition to RNA, RRM2 can interact with the amino acid sequence (S/G)(I/L)LGXXP present in the co-repressor proteins Raver1 and Raver2 [43–46], enabling PTB to recruit other regulatory proteins in AS events.

## 4. PTB Paralogs and Co-Factors

Two PTB paralogs are generally expressed in mammalian tissues: the nPTB/brPTB/PTBP2 which is expressed at high levels in adult brain, muscle and testis [47,48] and ROD1 (PTBP3) which is expressed preferentially in haematopoietic cells [49]. ROD1 has been demonstrated to act prevalently in the nonsense-mediated mRNA decay (NMD) of aberrant spliced isoforms through the interaction with key factors of the RNA surveillance pathway, such as the UPF1 factor, with which it shares the endogenous targets [50]. Rodents, but not humans, express an additional paralog, named smPTB, which is highly expressed in smooth muscle cells [51].

PTB and its paralogs share more than 70% amino acid sequence identity organized in the four RRM-type domains. The most extensively studied is the nPTB, initially described as a neuron specific splicing factor [52]. Further studies have shown that nPTB can be alternatively spliced, leading to different isoforms expressed in a tissue specific manner [48,53]. The expression of PTB and its paralogs is tightly regulated through alternative splicing events. PTB regulates its own expression by repressing the splicing of exon 11, causing NMD of the resultant mRNA [54], but it also promotes the exon 10 exclusion from nPTB transcripts, leading to NMD of nPTB transcripts. Both PTB and nPTB may promote the non-productive splicing of ROD1 [55]. In neuronal cells, nPTB acts by autoregulating its own exon 10 inclusion, leading to an increased expression level of the nPTB protein [56].

In T lymphocytes, both PTB and the alternatively spliced isoform lacking RRM1and RRM2, called PTB-T, participate in humoral and cellular immunity by targeting the CD154 cytokine mRNA. The activity is elicited in the cytoplasm by binding to pyrimidine *cis*-acting sequences at the 3′ untranslated region (3′UTR) of CD154. In activated T lymphocytes, the alternatively spliced isoforms of PTB expressed at different amounts act as *trans*-factors competing with each other for binding to the CD154 3′UTR, and modulating CD154 mRNA stability [57]. These data confirm that, although PTB is ubiquitously expressed, its activity is tissue specific and is mediated by the relative amount of alternatively spliced isoforms, paralogs, or competing factors for binding to polypyrimidine tracts.

As a splicing repressor factor, PTB may require the binding to selected factors that act as co-repressors. Raver1 and Raver2 are two well characterized PTB co-repressors [58–60]. Raver1 has been demonstrated to be essential for effective repressor function of PTB in the splicing repression of α-tropomyosin exon 3 [59,61]. A cooperative action in regulating adjacent exons has been confirmed by the structural model of PTB RRM2 bound to the Raver1 peptide [43]. The human *Raver*1 gene is ubiquitously expressed in tissues at different levels and encodes a 748 amino acid protein containing three RRMs, two nuclear localization signals (NLSs), one nuclear export sequence and a proline-rich region at its *C*-terminal region [58,62,63]. Similarly to PTB, Raver1 is a shuttle-protein. By analizing the interaction specificity, Raver1 has been demonstrated to interact with the cytoskeletal proteins α-actinin and metavinculin in the cytoplasm [58]. During myotube differentiation, Raver1 migrates to the cytoplasm, co-localizes with the microfilament attachment sites [58,63] and may be involved in the formation of adhesion complexes and intercalated discs in muscle cells [64]. In a *raver1* null mutant mouse, the loss of Raver1 is associated with a reduction of synaptic plasticity [65]. A recent report has shown that Raver1 may act also as a co-activator of the RIG-I-like receptor MDA5 (melanoma differentiation-associated gene 5), which is involved in the innate antiviral response [66].

Raver2, the additional PTB co-factor is a 625 aa protein of 72 kDa with a similar overall organization of the functional domains when compared to Raver1. Raver2 expression is restricted to differentiated neurons and glia cells. We have recently contributed to the characterization of *Raver1* and *Raver2* promoters and to the identification of *cis*-acting signals [62,67]. In contrast to Raver1, which binds to cytosolic proteins, so far there is no evidence of a Raver2 interaction with cytoplasmic proteins. PTB interacts with Raver2 by recognizing the SLLGEPP peptide motif, which is well conserved in both Raver1 and Raver2 [45].

## 5. PTB Role in Pre-mRNA Splicing

**Figure 3:**
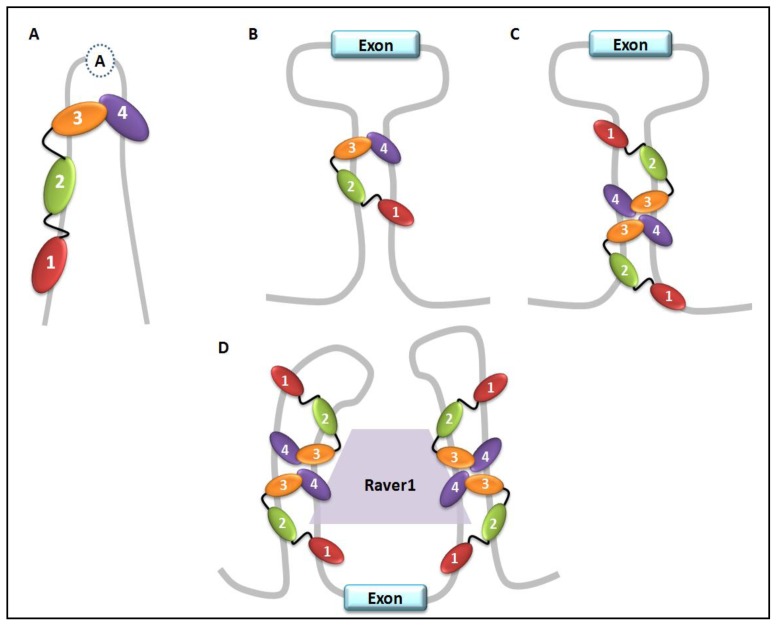
Models of RNA-PTB interaction in exon splicing repression. (**A**) Repression by PTB looping out a branch-point adenosine; (**B**) PTB monomer looping-out an exon; (**C**) Multiple PTB molecules binding cooperatively around an alternative exon; (**D**) PTB cooperates with the co-factor Raver1 to loop out an alternative exon by binding to distant intronic pyrimidine tracts.

PTB is implicated in the control of alternative exon selection during mRNA processing of many different transcribed genes, including its own pre-mRNA. The exon typologies include short alternative exons named “cassette exons”, “mutually exclusive exons” or alternative 3′ terminal exons [17,54,61,68] (summarized in Table 1). The polypyrimidine stretches recognized by PTB are frequently clustered in the intron sequences which surround the regulated exon, but they may also be present in the exon sequences. The most investigated mechanism of PTB action is related to its role in repressing the exon inclusion. To summarize the results derived from these studies, four different structural models may represent the dynamic of the different types of PTB splicing repression activity (Figure 3).

These models describe a looping-out RNA mechanism in which PTB binds distant pyrimidine tracts, resulting in a new position in which the polypyrimidine tracts are close to each other [17,68]. All models require multiple PTB binding sites positioned in the introns. The polypyrimide sequences may be at the branch-point adenosine or at intron sequences, both upstream or downstream to the regulated exon (Figure 3A,B). A slightly different RNA loop model requires the cooperative multimerization of PTB (Figure 3C) that involves RRM3 and RRM4 binding. The repression may occur by preventing the binding of spliceosomal components. These models usually involve the dimerization of PTB and the competition with the splicing factor U2AF65 for binding to polypyrimidine tracts. However, PTB does not always dimerize to repress splicing, as shown for the γ-aminobutyric acid gamma2 (GABA_A_-γ2) exon 9 regulation, in which a single PTB molecule is required to loop out a branch-point adenosine [87]. In the fourth model, the repression of exon inclusion derives by PTB interaction with sequences upstream and downstream of the regulated exon, building a scaffold structure that may recruit splicing auxiliary elements, such as Raver1 co-factor (Figure 3D).

Additional factors, other than U2AF65, may antagonize the PTB repressive role such as the ETR3-like factor (CELF) proteins CUG-BP, RBM4, TIA-1, Nova, and Fox-1 [68]. Recently, the muscleblind-like (MBNL1) protein that acts as a direct RNA-binding regulator of alternative splicing during development of striate muscle has been demonstrated to directly interact with PTB, promoting the skipping of α-tropomyosin exon 3 in smooth muscle cells. In this model, MBNL1 binding to RNA promotes the interaction with PTB, and consecutively, it induces an RNA conformational change [88].

Microarray-based genome wide approaches and CLIP-seq methods have shown that PTB not only acts as a splicing repressor, but can also enhance the inclusion of exons [89–92]. A global representation of the putative RNA targets containing the *cis*-acting splicing regulatory elements recognized by PTB has been derived by high-density oligonucleotide splice-sensitive microarray analyses in HeLa cells, where PTB was knocked down [93]. The results showed that the PTB-regulated splicing events were represented by both exon skipping and exon inclusion, and the majority of the events, almost 70%, were PTB-repressed exons’ inclusion. The exon inclusion determined by PTB may be attributable to the position of the PTB-binding motifs, upstream or downstream to the regulated exon [93]. Two different models have been suggested to describe the exon inclusion. One model proposes that PTB inclusion is activated through the binding to polypyrimidine stretches positioned exclusively downstream to the exon to be included [93]. The alternative model suggests that inclusion derives from binding to sequences near the flanking constitutive splice sites of the exon, competing with and antagonizing the action of different splicing repressors [92,94]. The data supporting the different PTB models of action converge on the common interpretation that PTB regulated splicing activities are consequences of a fine-tuning of the dynamic spatial, stoichiometrical PTB binding to RNA polypyrimidine motifs and of protein-protein interactions [95]. Although the mechanism of PTB activity is not completely understood, all models confirm the described motif recognition by PTB and its specific mRNA targets. Most of them have been utilized as validation targets for biophysical models to identify splicing regulatory elements and their interactions [86,96,97].

## 6. PTB Role in Internal Ribosome Entry Site (IRES)-Mediated Translation Initiation

PTB has the property to shuttle between nucleus and cytoplasm. Whereas in the nucleus PTB acts as an alternative splicing factor, in the cytoplasm it is involved in post-transcriptional regulation processes that require cap-independent translational controls, RNA localization and stability.

PTB participation in internal ribosome entry site (IRES)-mediated translation initiation of both cellular and viral mRNAs is extensively documented. IRESs require the formation of specific RNA structural conformations at the 5′UTR of the mRNA that help the recruitment of the translational machinery. Additional cellular factors, known as initiation of translation accessory factors (ITAFs), may bind the IRESs and lead to a more efficient translation initiation [98]. PTB has been frequently described as ITAF of IRES-mediated translation in human viruses including hepatitis viruses, several picornaviruses (alphaviruses and cardioviruses), flaviviruses such as hepatitis C (HCV), and noroviruses [99–101]. PTB and its paralog nPTB are key factors to allow the functional conformation of viral IRESs sequences [102,103], acting prevalently as mRNA chaperones [104]. In poliovirus infected cells, PTB is extensively re-localized to the cytoplasm, as a consequence of the proteolysis of the nuclear pore complex caused by the poliovirus.

PTB is a determinant of tissue and host tropism for rhinovirus and poliovirus [105–107]. PTB not only is involved in viral RNA translation but also in viral replication, as demonstrated for HCV [108] and dengue viruses (DENV) [109,110]. Interestingly, PTB may be cleaved during viral infection as a consequence of the shut off mechanism of host cell protein synthesis [105,111]. PTB plays the role of ITAF not exclusively in viral infected cells, but also in the translation of several host cellular factors, as listed in Table 2.

The mouse homologue of PTB is involved in the regulation of early mouse development through IRES dependent translation of CDK11 (p58) protein kinase and modulates the cell cycle in mouse embryonic stem cells [117]. In the context of inflammatory responsive gene expression regulation, PTB together with additional ITAFs targets the early growth response 2 (egr2) 5′UTR, enhancing IRES-dependent translation under proinflammatory conditions [118]. It has been demonstrated *in vitro* that PTB binds specifically to the insulin mRNA 5′UTR, when cap-independent insulin biosynthesis is produced in response to nitrosative stress [130]. PTB positively regulates the IRES activities of p53 isoforms, relocating them from the nucleus to the cytoplasm during stress conditions [119] and regulates the IRES activity of Cat-1 (cationic amino acid transporter) during nutritional stress [120].

As a chaperone, PTB influences apoptotic peptidase activating factor 1 (Apaf1) translation by recognizing the Apaf1-IRES structure [102] and positively regulates the translation of hypoxia-inducible factor 1 alpha (HIF-1 alpha) and cyclin-dependent kinase inhibitor 1B (p27Kip1) [121,122]. For most of the mRNAs, PTB positively regulates the IRES-mediated translation [131], but not always, as in the case of PTB effect on UNR (upstream *N*-ras) translation regulatory factor [129]. Cytoplasmic levels of PTB are increased by apoptosis or in response to toxic drugs, leading to modifications in the activity of selected IRESs containing transcripts [132,133].

## 7. PTB Role in RNA Polyadenylation, Transport and Stability

PTB has been found to be associated with pyrimidine-tracts present at 5′ and/or 3′UTRs of mRNAs. By binding to UTR sequences, PTB may be involved in the 3′-end processing, localization, and stability of several mRNAs [132,134–138]. In the neurite growth process, the export of PTB from the nucleus to neurite growth terminals has been associated with the localization of α-actin mRNA bound to PTB at the neurite terminals [136]. A similar role in facilitating mRNA export has been proposed for PTB as a co-factor in nuclear export of hepatitis B virus RNA by recognition of a post-transcriptional regulatory element [139]. Additional studies have highlighted the cytoplasmic role of PTB in the regulation of mRNA stability. PTB binding to pyrimidine-rich sequences downstream of the termination codon and upstream to the polyadenylation signal in preproinsulin mRNA determines increased preproinsulin mRNA stability and levels [140]. PTB promotes the stabilization of additional insulin granule components, such as islet cell autoantigen (ICA)512, prohormone convertases 1/3 (PC1/3), and 2 (PC2) mRNAs [141]. A similar effect of PTB on mRNA stability has been also demonstrated for vascular endothelial growth factor, HIF-1α and inducible NO synthase transcripts [126,135,142–144]. Stability of mRNA in activated T and B cells is regulated by a pathway that also involves PTB, as demonstrated for CD40 ligand and Rab8A [145–148]. PTB has also been found to be associated with the 3′UTR of the ATP synthase β-subunit, where it enhances translation in a cap-independent manner [149]. 5′-end processing efficiency is enhanced in the pre-mRNA of complement 2 (C2), protrombin (F2) and cyclooxygenase-2 (COX-2) [150–152], whereas PTB binding to the GU-rich sequences downstream to the polyadenylation signal, decreased the efficiency of 3′-end cleavage of the human α-globin and β-globin pre-mRNAs [134,137]. An interesting PTB role derives from the mouse model of circadian mRNA oscillation, in which PTB mediates the mRNA decay of expressed *mper2*, a circadian core clock gene, by binding to the 3′UTR CU-rich sequences [153].

An additional PTB functional role that deserves to be highlighted derives from the studies on *Drosophila* and *Xenopus* development. These studies demonstrate that PTB plays a role in the remodeling of RNP, promoting RNA localization and translation regulation of critical determinants of the embryo development. In the cytoplasm, *Drosophila* PTB, known as the product of the *hephaestus* locus, binds to multiple sites in the *oskar* 3′UTR leading to mRNA oligomerization that causes *oskar* translation repression. The model proposed in this study is that PTB may promote the formation of densely packed RNP particles with *oskar* RNAs, thereby preventing access for the translation machinery [154]. A similar role in RNP remodeling has been attributed to the *Xenopus* PTB ortholog. In *Xenopus* oocytes, PTB is required for transport and localization of the Vg1 RNA, a critical factor for the correct patterning of the embryo. Lewis *et al.* [155] have demonstrated that PTB is associated with RNP complexes including Vg1 RNA in the nucleus and in the cytoplasm. During cytoplasmic translocation PTB promotes the remodeling of the Vg1 RNP by modulating protein-protein interaction within the Vg1 RNP.

## 8. PTB Role in Cell Type Differentiation

Based on the extensive literature supporting the relevance of PTB in multiple biological processes, including development, as mentioned in section seven for germ cell differentiation in *Drosophila* and in development of *Xenopus* [154,155], it is not surprising to find evidence for an essential role of PTB also in mammalian development. In fact, in *ptb* knockout mice the absence of PTB results in embryonic lethality and severe defects in proliferation and differentiation of embryonic stem cells [156]. As well, a key role of PTB in cell differentiation is revealed by recent studies on muscle and neuronal cell differentiation [86,157]. Wide gene expression analyses demonstrate that PTB and nPTB expression is associated with repression of various neuronal and striate muscle specific exons in genes encoding cytoskeletal components and proteins involved in cell signalling [55,92,93]. Both PTB and nPTB protein levels decrease during myogenesis, affecting exon splicing of several transcripts regulated during myoblast differentiation [89]. Splicing-sensitive microarray analyses have demonstrated a combinatorial control of the myogenic splicing program by PTB and additional proteins that may compete for RNA binding. This is the case for the Quaking (QK) protein whose expression is raised during differentiation of myoblasts to myotubes, whereas PTB levels are reduced [158]. An estimation of the exons regulated by both QK and PTB, indicates that nearly 39% of the QK splicing network is also controlled by PTB [156]. An interesting model of PTB action in competition with an additional splicing regulatory factor during muscle cell differentiation, has been proposed by Lin and Tarn [159,160]. The authors demonstrate that the RNA binding motif 4 protein (RBM4) competes for common targets with PTB and nPTB, antagonizing their effects in muscle cell-specific alternative splicing. RBM4 contributes, at least in part, to the reduction of PTB and nPTB levels during the C2C12 cells differentiation, involving an AS-NMD pathway by activating exon skipping of PTB/nPTB trancripts. PTB contributes to post-transcriptional regulation of gene expression also during cardiomyocyte differentiation. As well as in myoblast differentiation, PTB expression is reduced in heart development. In differentiating cardiomyocytes, PTB regulates the expression of apoptotic genes [161]. Ye *et al.* [84] have demonstrated the direct involvement of PTB in the AS regulation of transcription factor myocyte enhancer-factor 2 (MEF2) and the cardiac structural proteins tropomyosin 1 (TPM1) and tropomyosin 2 (TPM2) during cardiomyocyte differentiation. *In vivo* and *in vitro* results demonstrate that the reduced expression of PTB derives from a caspase-dependent cleavage of the protein during the myocardium development. The process is regulated by histone deacetylases and the caspase inhibitor FLICE-like protein (cFLIP), which induce the PTB degradation during cardiac muscle differentiation.

**Figure 4:**
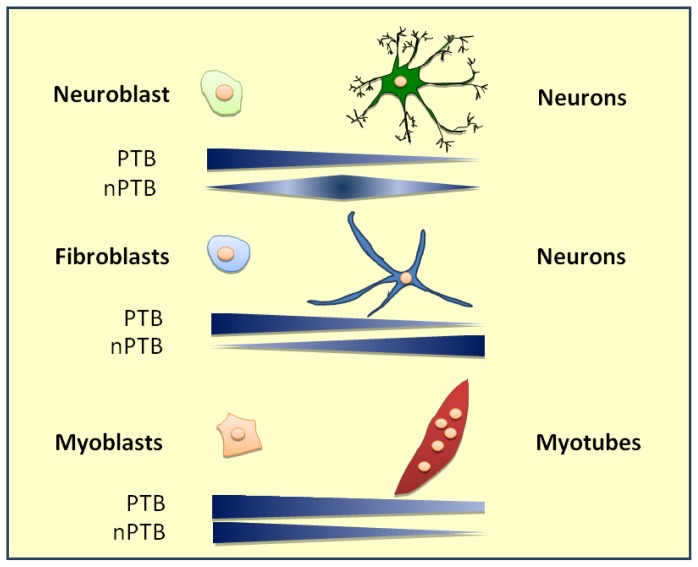
Relative ratio of PTB and nPTB expression during cell differentiation.

A relevant role played by PTB in splicing regulation during cell differentiation is highlighted by studies on neuronal differentiation, reviewed in [18]. PTB and its homolog nPTB are subject to a programmed switch during neuronal development [89,162]. In post-mitotic neurons a reduced expression of PTB coincides with an increased expression of nPTB, which modifies the neuronal alternative splicing program [47,52,90,163]. A similar switch in the expression of PTB *versus* nPTB seems to occur in post-mitotic lens fibre cells [164]. A recent study described by Zheng *et al.* [86] in a mouse model reveals new clues for the understanding of the PTB role during neuronal development. The authors describe three defined phases during neuronal differentiation in which different levels of PTB and nPTB expression mediate splicing regulation. These three phases are characterized by the expression of PTB regulated alternative splice isoforms of the postsynaptic density protein 95 (PSD-95). PSD-95 is an essential factor required for synaptic maturation and plasticity. Both PTB and nPTB repress PSD-95 exon 18 inclusion, leading to premature translation termination and subsequent NMD of the spliced isoform. The reduced expression of PSD-95 protein prevents a precocious stabilization of early generated synapses. The results show that during the initial phase occurring in neuronal stem cells, PTB expression is associated with low levels of PSD-95. The transition to the second phase, represented by the start of neuronal differentiation, comes along with the sequential shift to an increased expression of nPTB and downregulation of PTB, but maintaining a sufficient amount of the two proteins to repress the PSD-95 protein expression. Finally, in the third phase, after mouse birth in cells undergoing synaptic maturation, both PTB and nPTB expression is reduced, causing the stability of the PSD-95 splicing isoforms and consequently an increase of the protein levels and the start of synaptic maturation. Further proof of the functional role of the switch in PTB/nPTB expression in controlling alternative splicing programs required during neuronal development is given by recent results showing the conversion of PTB-depleted fibroblast into neuronal-like cells [165]. A comprehensive view of the regulated expression of PTB and nPTB during cell differentiation is presented in Figure 4.

## 9. PTB Regulation Mediated by miRNA

The levels of PTB and nPTB expression are subject to microRNA control. Given the estabilished role of microRNAs (miRNAs) in post-transcriptional gene expression regulation, their involvement in PTB and nPTB expression during cell differentiation was expected to be demonstrated. A number of pieces of evidence are now available supporting the function of tissue specific microRNAs in the regulation of PTB expression. The reduced level of PTB and the almost complete absence of nPTB during myoblast differentiation, have been demonstrated to be associated with the induction of the muscle-specific microRNA miR-133 expression [89]. During neurogenesis PTB is downregulated by the activity of the neuron specific microRNA miR-124 [162]. MicroRNAs roles as regulators of PTB levels in specific tissue contexts have also been supported by studies in human pancreatic islets responding to the presence of high levels of glucose. MiR-133a is induced by high amounts of glucose leading to lower PTB levels and lower insulin biosynthesis rates by the effect of mRNA destabilization [166]. Xue *et al.* [165] demonstrate the power of PTB in reprogramming the differentiation of fibroblast cells types into neuronal-like cells, involving the activity of PTB regulated microRNA circuits. The stable knockdown of PTB obtained by small hairpin RNAs in fibroblast cells leads to the switch to neuronal-like cells. Thus, the absence of PTB leads to the reprogramming of non-neuronal cells into neuronal types by removing a PTB mediated block of microRNAs action. The authors showed that miR-124 represses neuronal genes in non-neuronal cells by an autoregulatory loop mechanism. PTB may positively or negatively regulate the microRNA targeting by two alternative actions. One activity is obtained by competing with microRNA targeting the same transcripts, the other one by promoting microRNA targeting additional transcripts and altering the local RNA structures. Engels *et al.* [167] have shown that PTB interacts with miRNAs and human Argonaute 2 (Ago2) in the RNA induced silencing complex. Their results showed that miRNAs may be co-regulated by PTB and Ago2 in human cells. We expect that in future studies analysing the global expression in PTB downregulated-reprogrammed cells, new interactions between PTB, additional RBPs and microRNA programs will be revealed.

## 10. PTB and Cancer

PTB expression may affect cancer initiation and progression [168,169]. Perinucleolar compartments (PNCs) that get formed in malignant cells during transformation, are enriched with RNA polymerase III RNAs and RNA binding proteins, including PTB [170]. PTB differentially affects cancer cell malignancy, depending on the cell type [169]. Increased levels of PTB expression have been associated with growth and maintenance of transformed properties of ovarian tumor cells [168,171]. Suppression of PTB expression by siRNA in ovarian cancer cells leads to inhibition of cell proliferation, anchorage-dependent growth, and cell invasion [168]. PTB may affect malignant cell growth by inducing aberrant splicing events in transcripts involved in tumor progression. PTB in fact induces exon skipping in genes that are involved in proliferation (*FGFR1*, *FGFR2*), apoptosis (*FAS*, *CASP2*), invasion (*CSRC*), motility (*ACTN, FBN*), and multi-drug resistance (*ABCC1*). In the cytoplasm, PTB increases IRES-mediated translation or RNA stability of transcribed genes involved in proliferation (*IGFR1*), angiogenesis (*VEGF*), and apoptosis (*APAF1*) [102,126–128]. The relevance of the PTB involvement in post-transcriptional regulation of key factors in tumor progression has been recently supported by the demonstration of the effect of a single nucleotide polymorphism (SNP) in the 5′UTR of the p53 tumor suppressor gene expressed in human melanoma tumors. In the cytoplasm PTB positively regulates the translation of p53 mRNA and its *N*-terminal truncated isoform by binding to IRESs *cis-*acting elements [119]. The presence of the SNP leads to a weaker binding to PTB and thereafter to a reduced cap-independent translation of p53 [172].

PTB forms complexes with transcripts encoding focal adhesion scaffolding proteins which may affect cell spreading [173,174]. Knockdown of PTB in glioma cell lines modifies the alternative splicing of the transmembrane RNT4 (reticulon 4) factor and decreases cell proliferation and migration, whereas cell adhesion is increased [175]. Recently, it has been reported that PTB promotes the switch of expression of two forms of pyruvate kinase, PKM1 and PKM2, resulting in an increase of the aerobic glycolysis and consequently in a rapid proliferation of cancer cells [176–178]. PTB, together with hnRNPA1 and hnRNPA2, acts in regulating the inclusion of exon 10 and the repression of exon 9 of the pyruvate kinase, resulting in the PKM2 isoform. In human gliomas PTB is overexpressed and the highest level of the protein correlates with the level of PKM2 expression [178]. An opposite effect of PTB overexpression has been demonstrated in the growth of non-small-cell lung cancer cell types. PTB inhibits the proliferation of H1299 and A549 cells and induces the expression of cyclin-dependent kinase inhibitors in H1299 cells [179]. In cells derived from patients with multiple myeloma, c-myc overexpression correlates with an increased interaction of PTB with factor YB-1, as well as with an increased expression of both factors, supporting the role of PTB in stimulating IRES-mediated translation [180]. Taken together, mis-regulation of PTB could cause multiple epigenetic changes in gene expression promoting tumorigenesis, which might be tissue-dependent.

## 11. Conclusions

The contributions derived from the intense studies on RNA binding proteins confirm that the unique properties of PTB RRM domains are the key elements enabling its multiple cellular functions. The relevant role played by PTB in most post-transcriptional processes is indeed based on its ability to recognize specific RNA sequences, modifying the local RNA structural arrangements. Competition in RNA binding between PTB and other regulators of the RNA metabolism appears critical to its diverse functions. As a consequence, the levels of PTB expression as well as the rate of nuclear import and export may become censorious to the cell fate. Thus, while the identification of new PTB targets is rapidly progressing by means of genome-wide investigations [92], an effort is now being made to identify chemical PTB modulators [181]. It has been demonstrated that tannic acid induces the expression of *PTB* by activating its promoter region. In congenital myasthenic syndrome, the aberrant inclusion of muscle nicotinic acetylcholine receptor α subunit (*CHRNA1*) exon P3A is caused by an intronic mutation. PTB overexpression by tannic acid has been demonstrated to foster *CHRNA1*exon P3A skipping, opening new possibilities of therapeutic approaches based on PTB expression regulation [182]. We expect that on-going and future studies on PTB activities will reveal additional aspects of the complex molecular machinery underlying tissue differentiation events, with a particular focus on the concurrent roles played by miRNAs.

## Figures and Tables

**Table 1:** Exons regulated by PTB.

Gene	Regulated Exon	Exon typology	Position of PTB-*cis*-acting elements	Ref.
*α-actinin*	SM	Mutually exclusive	Upstream	[69,70]
*α-actinin*	NM	Mutually exclusive	Flanking the branch-point	[69,70]
*α-tropomyosin*	3	Cassette	Up- and downstream	[71]
*ATP syntetase γ-subunit*	9	Cassette	Upstream	[72]
*β-tropomyosin*	7	Mutually exclusive	Upstream	[73]
*c-src tyrosine kinase*	N1	Cassette	Up- and downstream	[74,75]
*Calcitonin*	4	Cassette	Downstream	[76]
*Caspase-2*	9	Cassette	Downstream	[77]
*Cardiac troponin T*	5	Cassette	Up- and downstream	[78]
*Clathrin light chain B*	EN	Cassette	Upstream	[79]
*FGF-R1*	α	Cassette	Up- and downstream	[80]
*FGF-R2*	IIIb	Mutually exclusive	Downstream	[81]
*GABA_A_γ2*	2	Cassette	Upstream	[79,82]
*IgM*	M1 M2	Cassette	Exonic	[83]
*NMDA receptor 1*	5	Cassette	Upstream	[79]
*MEF2*	β	Cassette	Up- and downstream	[84]
*CaV1.2 calcium channel*	8a-8	Mutually exclusive	Upstream	[85]
*PSD-95*	18	Cassette	Upstream	[86]
*PTB*	11	Cassette	Exonic	[54]

**Table 2:** IRESs interacting with PTB.

mRNA	PTB effect in facilitating translational initiation	Ref.
HAV	+	[112]
EMCV	+	[42]
Poliovirus	+	[113]
HVC	+	[108]
Norovirus	+	[114]
DENV	+	[115]
FMDV	+	[116]
CVB3 (coxsackievirus B3)	+	[100]
CDK11 (p58)	+	[117]
EGR2	+	[118]
INSULIN	+	[103]
p53	+	[119]
Cat-1	+	[120]
APAF-1	+	[102]
HIF-1alpha	+	[121]
p27Kip1	+	[122]
IRF2	+	[123]
Rev-erb α	+	[124]
c-myc	+	[125]
VEGF	+	[126,127]
IGFR1	n.d.	[128]
IR (insulin receptor)	+	[103]
UNR	−	[129]
